# The loading impact of training and match-play on non-contact muscle injuries in elite male soccer players. A seasonal analysis

**DOI:** 10.5114/biolsport.2026.153305

**Published:** 2025-08-06

**Authors:** Ryland Morgans, Rafael Oliveira, Mauro Mandorino, Piotr Zmijewski, Ben Ryan, Toni Modric, Jose Teixeira, Alexandre Moreira

**Affiliations:** 1 School of Sport and Health Sciences, Cardiff Metropolitan University, Cardiff, UK; 2 Brentford FC Football Research Centre, Brentford FC, London, UK; 3 Research Centre in Sport Sciences, Health Sciences and Human Development (CIDESD), Santarém Polytechnic University, 2040-413 Rio Maior, Portugal; 4 Santarém Polytechnic University, School of Sport, Av. Dr. Mário Soares, 2040-413 Rio Maior, Portugal; 5 Performance and Analytics Department, Parma Calcio 1913, 43121 Parma, Italy; 6 Department of Movement, Human and Health Sciences, University of Rome “Foro Italico”, 00135 Rome, Italy; 7 Jozef Pilsudski University of Physical Education in Warsaw, 00-809 Warsaw, Poland; 8 Research and Development Center Legia Lab, Legia Warszawa, Poland; 9 Faculty of Kinesiology, University of Split, Split, Croatia; 10 High Performance Sport Center, Croatian Olympic Committee, Zagreb, Croatia; 11 Research Centre in Sports, Health and Human Development, Covilhã, Portugal; 12 Department of Sport Sciences, Instituto Politécnico de Bragança, Bragança, Portugal; 13 Department of Sport, School of Physical Education and Sport, University of São Paulo, São Paulo, Brazil

**Keywords:** External load, Football, Non-contact muscle injuries, Sprinting, High-speed running, Soccer

## Abstract

This study investigated the relationship between acute and chronic training load metrics and non-contact muscle injuries in elite soccer players employing a novel statistical approach. A retrospective analysis was conducted during the 2020/21 season on 30 senior outfield players from an English Premier League club. Global Positioning System (GPS) technology monitored total distance, high-speed running (HSR) distance (5.5–7 m/s), sprint distance (> 7 m/s), and peak speed during training sessions and matches. A total of 42 injuries were documented, with an incidence of 8.94 injuries per 1000 hours, although only 12 non-contact muscle injuries were included in the analysis that occurred at 2.5 per 1000 hours of exposure. Acute (7-day) and chronic (28-day) training loads were examined, and data preprocessing addressed missing values and multicollinearity. To address class imbalance, the dataset was balanced using the Synthetic Minority Over-Sampling Technique (SMOTE) prior to logistic regression. Four significant predictors were retained: acute HSR (β = -0.175, *p* < 0.001), acute sprint distance (β = -0.613, *p* < 0.001), acute peak speed (β = 1.101, *p* < 0.001), and chronic total distance (β = 2.234, *p* < 0.001). The model demonstrated excellent discriminative ability with an AUC-ROC of 0.80. The results showed that higher acute volumes of HSR and sprint distance serve as protective factors against non-contact muscle injuries, whereas an increase in acute peak speed and chronic total distance significantly elevates injury risk. These findings underscore the importance of regular exposure to HSR to enhance injury resilience, while excessive load and peak speed may contribute to neuromuscular fatigue and overload.

## INTRODUCTION

The most decisive moments during a soccer match include highintensity actions such as sprinting, jumping and changes of direction [1] and are associated with scoring a goal [2]. Some of these actions can be monitored by a global positioning system (GPS), local positioning systems (LPS), or inertial measurement units that belong to micro-electro-mechanical systems [3]. When these systems are combined to monitor both internal and external loads with an athlete recovery measurement, a better understanding of the training stimulus and individual response is achieved. Thus, allowing an appropriate training load to be implemented aiming to minimize injury risk [4]. According to a previous study in professional male soccer, there are 8.1 injuries per 1000 hours of training/match exposure [5]. The ratio is higher when only matches are considered (36 injuries per 1000 hours of exposure) compared to training sessions (3.7 injuries per 1000 hours of exposure) [6].

In soccer, high-speed running (HSR) and sprinting demands are associated with 70% of non-contact muscle injuries [5]. For instance, Jiménez-Rubio et al. [7] analyzed the differences in highintensity distances and maximum velocity sprints comparing pre- and post-injury data and found that only slight improvements in these measures were observed when the player returned to play. However, this study did not investigate the loading patterns of highintensity running and sprinting prior to injury occurrence or pre- and post-injury performance [7]. Furthermore, Morgans et al. [8] and Raya-González et al. [9] have attempted to analyze the impact of all injuries on match running performance following the return to competitive match-play over two consecutive seasons. However, none of these previous studies examined training and/or match load during a specific period prior to injury. Although Whiteley et al. [10] analyzed HSR performance during match-play prior to and following a hamstring injury in the Qatar Stars League, where the main findings reported that when players returned to matchplay post-hamstring injury, reductions in HSR during match-play were observed [10].

Additionally, there are some indexes that allow more detailed analysis regarding the impact of the physical demands and the variation between weeks such as acute (total load of the last 7 days) and chronic loads (total load of the last 21 or 28 days) [11, 12]. Some studies analyzed the relationship of these indexes with hamstrings injuries [13, 14]. Gee et al. [14] found that most hamstring injuries occurred when the mean acute:chronic workload ratio (ACWR) was below the squad mean ACWR for the season. The loading pattern of ‘moderate to high’ followed by ‘low to moderate’ ACWR was commonly observed in the four weeks prior to injury [14]. While Ribeiro-Alvares et al. [13] found that 75% of the analyzed players had ACWR below 1.5 for total distance, HSR distance, very high-speed running distance, and the number of actions performed > 5.3 m/s, immediately before the training session or match in which the hamstring injury was sustained.

Considering the scarce knowledge of analyzing acute and chronic loads prior to injury occurrence, the aim of this study was to examine the relationship between total distance, HSR, sprint distance and non-contact muscle injury during a 7-day (acute) and 28-day (chronic) period across one season in an English Premier League soccer club. Specifically, the aim was to investigate the rolling sum of total distance, HSR, sprint distance and the peak speed achieved in the acute and chronic periods preceding non-contact muscle injuries by comparing injured and uninjured players employing a novel statistical approach. Considering previous research, the study hypothesis was that increased acute and chronic loads may be associated with higher injury occurrence [13].

## MATERIALS AND METHODS

A retrospective analysis was conducted utilizing player injury, training and match running data collected in an elite English Premier League soccer team. A club physiotherapist consistently recorded availability and injury data daily in a standardized format for each player. Previous definitions and data collection procedures in soccer injury studies [15, 16] were implemented in this study. Players that were previously injured at study commencement were included, although were not incorporated into the analysis.

This research utilized a one-year longitudinal study design. A nonprobabilistic sampling protocol was employed to recruit the participants. During the observational period of season 2020/21, consistent player monitoring approaches were practically implemented by club staff without any interference from the researchers.

### Participants

Thirty professional outfield soccer players from an English Premier League club were involved in the study. Data from the complete 2020/21 season included 30 senior players (first-team squad) (age 24.2 ± 6.1 years, weight 74.7 ± 7.8 kg, height 1.81 ± 0.09 m). The inclusion criteria for the study have previously been applied [17, 18, 19] and were: (i) listed on the roster of the first-team squad at the start of the study season, (ii) regularly trained with the first-team, (iii) participated in at least 80% of training sessions and matches, and (iv) did not participate in another training program during the study. Additionally, the exclusion criteria for the study have also been applied [20, 21] and included: (i) long-term (three months or longer) injured player, (ii) joining the team late in the study season, (iii) lack of full, complete data for training or match-play, (iv) an in-sufficient number of satellite connection signals, and (v) goalkeepers, due to the variations in running demands.

Players were classified as: centre-backs (CB; n = 7), full-backs (FB; n = 4), centre midfielders (CM; n = 9), attacking midfielders (AM; n = 5), and centre forwards (CF; n = 5). If a player fulfilled multiple playing positions during match-play, the player was categorized accordingly to each position. Players with history of previous non-contact injuries at study commencement were included in the analysis although not specifically analyzed.

All data collected resulted from normal analytical procedures regarding player monitoring over the study season, nevertheless, written informed consent was obtained from all participants. The study was conducted according to the requirements of the Declaration of Helsinki and was approved by the local Ethics Committee of Cardiff Metropolitan University and the professional club from which the participants volunteered [22]. To ensure confidentiality, all data were anonymized prior to analysis. All players provided consent for injury-related and match running performance data to be collected and utilized for research purposes.

All injuries recorded in outfield players from the study team across the 2020/21 season were considered. Among the 42 injuries, 12 different non-contact muscle injuries were considered for the analysis.

### Data collection

The initial phase of the season was classified as pre-season (early-July to mid-August) which lasted five to six weeks, and the competitive season (mid-August to mid-May) that fulfilled 36 weeks. Players’ external load was recorded during 234 training sessions and 68 official matches. An average of 168 ± 45 observations per player were recorded.

All injuries were assessed, diagnosed, and categorized by the club’s physiotherapist and head doctor and defined as injury incidences resulting in a modified training program, or missed training sessions or matches [8, 23]. Injuries were further categorized by body-site (injury location and region), injury type (trauma, overuse), sub-type (fracture, other bone injury, dislocation/subluxation, sprain/ligament, meniscus/cartilage, concussion, muscle strain, tendon, hematoma/contusion, nerve injury and synovitis), nature (training, match or undefined), description and days missed [15]. However, all noncontact muscle injuries were initially diagnosed by clinical assessment and clarified by imaging confirmation and classification system was used to determine the severity. Only non-contact muscle injuries were considered for analysis.

Physical data were also consistently monitored across the study season during all training sessions and matches using an 18 Hz Global Positioning System (GPS) technology tracking system (Apex Pod, version 4.03, 50 g, 88 × 33 mm; Statsports; Northern Ireland, UK). This Apex Pod system has been previously examined utilizing a between-analysis (t-test) for total distance and peak speed and reported non-significant differences and reported a small error of around 1–2% compared to the criterion distances during 400 m, 128.5 m circuit, 20 m trials and peak speed in a student population [24]. All devices were activated 30-minutes before data collection to allow the acquisition of satellite signals and to synchronize the GPS clock with the satellite’s atomic clock [25]. Quantifying the devices’ accuracy when determining average speed indicated improving accuracy as the distance covered increased and the speed of movement decreased (bias, %; 5 m: 9 ± 6%; 20 m: 3 ± 3%) [26]. To avoid potential inter-unit variation players wore the same GPS unit for each training session and match [27], although the present GPS system has previously reported excellent inter-unit reliability [28]. The GPS signal quality and horizontal dilution of position (HDOP) was connected to a mean number of 21 ± 3 satellites, range 18–23, while HDOP for all seasons ranged between 0.9–1.3.

On completion of each training session or match, GPS data were extracted and processed prior to analysis using proprietary software (Apex, 10 Hz version 4.3.8, Statsports Software; Northern Ireland, UK) as software-derived data is a more simple and efficient way for practitioners to obtain data in an applied environment, with no differences reported between processing methods (software-derived to raw processed) [27]. The dwell time (minimum effort duration) was set at 0.5 s to detect HSR and 1 s to detect sprint distances, in-line with manufacturers recommendations and default settings to maintain consistent data processing [27]. Furthermore, the internal processing of the GPS units utilized the Doppler shift method to calculate both distance and velocity data which is shown to display a higher level of precision and less error compared with data calculated via positional differentiation [29].

The following training load metrics were examined:

– Acute Total Distance (A-TD): Rolling sum of total distance covered within a 7-day period.
– Acute High-Speed Running (A-HSR) (5.5–7 m/s): Rolling sum of high-speed running distance within a 7-day period.
– Acute Sprint Distance (A-SD) (> 7 m/s): Rolling sum of sprint distance within a 7-day period.
– Acute Peak Speed (A-PS): Maximum speed achieved in the last seven days.
– Chronic Total Distance (C-TD): Rolling sum of total distance covered within a 28-day period.
– Chronic High-Speed Running (C-HSR): Rolling sum of high-speed running distance within a 28-day period.
– Chronic Sprint Distance (C-SD): Rolling sum of sprint distance within a 28-day period.
– Chronic Peak Speed (C-PS): Maximum speed achieved in the last 28 days.

### Statistical analysis

The statistical analysis aimed to examine the relationship between training load metrics as independent variables and non-contact muscle injuries as the dependent variable, categorized as 0 (no injury) and 1 (non-contact muscle injury). Prior to analysis, missing values in numerical variables were imputed using the mean of the respective columns. To address multicollinearity, a correlation matrix was computed, and variables with a correlation coefficient above 0.8 were eliminated. Additionally, the Variance Inflation Factor (VIF) was calculated, and variables exceeding a VIF of 10 were excluded. The remaining variables were standardized using z-score normalization to ensure comparability. Given the imbalance between injury and non-injury cases, the Synthetic Minority Over-Sampling Technique (SMOTE) was applied, combining under-sampling of the majority class and over-sampling of the minority class by generating synthetic observations to balance the dataset [30, 31]. After data pre-processing, a logistic regression model was employed to predict injury risk, utilizing the “saga” solver with a maximum of 3000 iterations to ensure convergence. Odds ratios (ORs) were calculated to quantify the effect of each predictor, and model performance was assessed using the Area Under the Receiver Operating Characteristic Curve (AUC-ROC), which measures the ability to distinguish between injured and non-injured players. The AUC-ROC values were interpreted as follows: 0.50 indicates no discrimination, 0.51–0.69 poor discrimination, 0.70–0.79 acceptable discrimination, 0.80–0.89 excellent discrimination, and values above 0.90 are considered outstanding [32].

## RESULTS

**FIG. 1:**
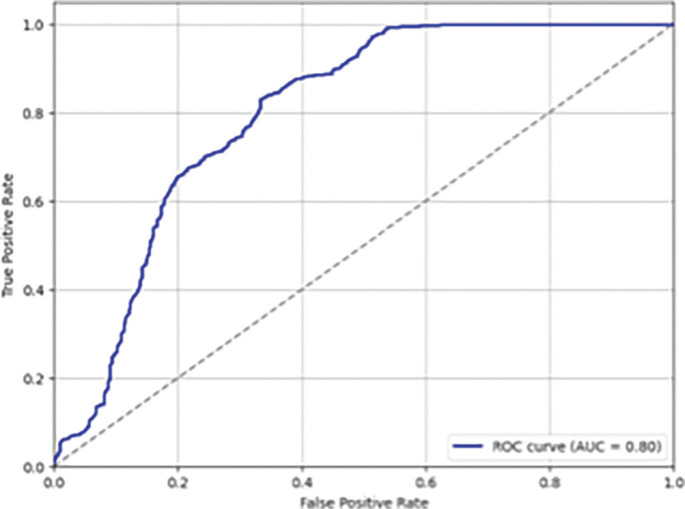
Estimation of logistic regression model performance using the receiver operating characteristic curve (AUC-ROC).

**FIG. 2:**
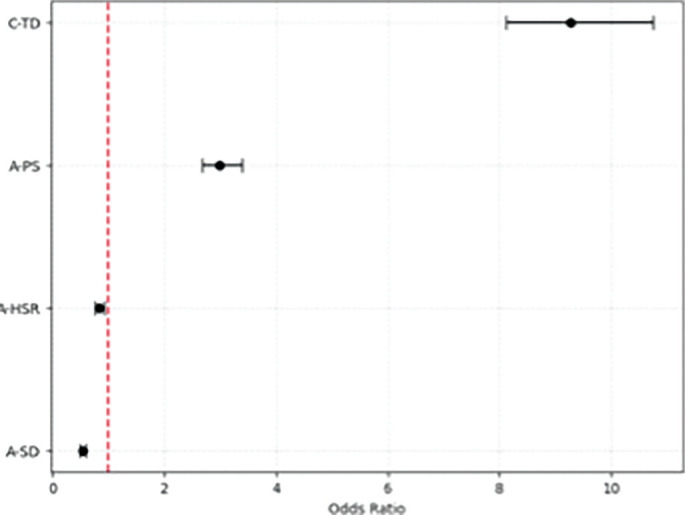
Forest Plot of the ORs with confidence intervals (95% CI).

During the analyzed season, 42 injuries were recorded, with an overall incidence of 8.94 injuries per 1000 hours of exposure. Among these, 12 injuries were classified as non-contact muscle injuries, with an incidence of 2.5 per 1000 hours and were included in the final logistic regression. Ten were recorded during official matches, while two during training. Seven recorded injuries involved hamstrings, three adductors, one soleus, and one peroneus muscle. The mean and standard deviation (SD) of number of absent days was 12.7 ± 16.9, with a range of 1 to 63. Mean and standard deviation of the variables included in the logistic regression are presented in Table 1 and grouped according to the dependent variable (no-injury, injury). Following feature selection and data balancing, four variables were retained in the final logistic regression model: A-HSR, A-SD, A-PS, and C-TD. The model demonstrated an AUC-ROC of 0.80, highlighting an excellent discriminatory ability (Figure 1). The logistic regression results were detailed in Table 2, while the ORs were plotted with confidence intervals in Figure 2, using a forest plot. The β coefficients and the ORs suggest that A-HSR and A-SD were identified as protective factors to reduce the risk of non-contact muscle injuries, while an increase in the A-PS, and C-TD were associated with an increased risk of injury.

**TABLE 1:** Mean ± standard deviation of the independent variables used for the logistic regression.

Variable	No-Injury condition	Injury condition
A-TD (m)	25009 ± 7831	27712 ± 5919
A-HSR (m)	1727 ± 739	1701 ± 558
A-SD (m)	173 ± 117	183 ± 86
A-PS (m/s)	8.8 ± 0.7	9.1 ± 0.5
C-TD (m)	87942 ± 29933	112107 ± 16100
C-HSR (m)	6117 ± 2205	7075 ± 1559
C-SD (m)	607 ± 319	723 ± 262
C-PS (m/s)	9.2 ± 0.7	9.5 ± 0.5

Note: A-TD: Acute Total Distance – the total distance covered over a 7-day period; A-HSR: Acute High-Speed Running Distance – the running distance covered at speeds between 5.5 and 7 m/s over a 7-day period; A-SD: Acute Sprint Distance – the running distance covered at speeds above 7 m/s over a 7-day period; A-PS: Acute Peak Speed – the maximum speed achieved during the last 7 days; C-TD: Chronic Total Distance – the total distance covered over a 28-day period; C-HSR: Chronic High-Speed Running Distance – the running distance at speeds between 5.5 and 7 m/s over a 28-day period; C-SD: Chronic Sprint Distance – the running distance covered at speeds above 7 m/s over a 28-day period; C-PS: Chronic Peak Speed – the maximum speed achieved during the last 28 days; au, arbitrary units.

**TABLE 2:** Logistic regression and odds ratio results.

Variables	Β co-efficient	Std. Error	p-value	95% CI
Intercept	-1.424	0.059	< 0.001	(-1.540, -1.309)
A-HSR	-0.175	0.055	< 0.001	(-0.282, -0.068)
A-SD	-0.613	0.056	< 0.001	(-0.723, -0.504)
A-PS	1.101	0.060	< 0.001	(0.984, 1.219)
C-TD	2.234	0.072	< 0.001	(2.094, 2.375)

Note: A-HSR: Acute High-Speed Running Distance – the running distance covered at speeds between 5.5 and 7.0 m/s achieved during the last 7 days; A-SD: Acute Sprint Distance – the total distance covered at speeds above 7.0 m/s achieved during the last 7 days; A-PS: Acute Peak Speed – the maximum speed achieved during the last 7 days. C-TD: Chronic Total Distance – the total distance covered over a 28-day period.

## DISCUSSION

The aim was to investigate the acute and chronic load of total distance, HSR, sprint distance and peak speed in periods preceding non-contact muscle injuries by comparing injured and uninjured players. The main findings showed that a higher volume of A-HSR and A-SD in the last seven days (acute load) significantly reduces the likelihood of non-contact muscle injuries, supporting the idea that regular exposure to high velocities may enhance injury resilience [33]. However, excessive exposure to peak speed within a short period (acute 7-day period) appears to elevate non-contact muscle injury risk, likely due to increased neuromuscular demand and fatigue. Indeed, previous studies suggested that moderate exposure to maximum velocity and high chronic training loads may reduce the risk of lower limb injuries in team sport players [34, 35]. Thus, the study hypothesis was partially supported as an increase in A-PS, and C-TD were associated with an increased risk of injury [13]. Nonetheless, an increase in A-HSR and A-SD were considered protective factors to reduce the risk of non-contact muscle injuries. Therefore, the relationship between load and injury is not universally linear, while the type of load and intensity specificity could further explain the load-injury relationship.

The present findings showed a tendency of higher non-contact muscle injury risk when excessive exposure to peak speed occurred (acute 7-day period), which may be attributed to higher neuromuscular demands and fatigue. In earlier studies, greater values of accelerometer-based variables have been related to neuromuscular fatigue, which may increase the likelihood of injury [36]. For instance, accelerations have a higher metabolic cost [37], while decelerations have a higher mechanical load [38]. These types of actions can produce greater damage on soft tissue structures due to the association with higher loads and force impact [39]. However, when these actions are not appropriately considered, monitored and utilized in match-play preparation methods, a higher risk of fatigue and injury may occur [40]. Nonetheless, actions like accelerations often precede a sprint or a peak speed action and therefore, may help justify the findings of the present study, although future studies should also include both accelerations and deceleration for further insights. Regarding peak speed and its relationship with injury, Malone et al. [35] reported that Gaelic football players with moderate exposures to maximum velocity (> 6–10) were at reduced injury risk compared to players who experienced a lower number (< 5) of exposures, when analyzing the previous four weeks [35]. Similarly to the present results, Malone et al. [34] reported that elite soccer players were at an increased risk of injury when exposed to high one-week cumulative training loads (1500 to 2120 au calculated by rating of perceived exertion). Although, when previous training load and intermittent aerobic fitness were considered, players with higher chronic loads (≥ 2584 au, calculated by rating of perceived exertion) completed greater HSR and sprint distances at a lower risk of injury [34] which may be associated with higher peak speed data. Notably, a high chronic load of total distance was identified as a risk factor, with an odds ratio above 1, indicating a potential overload effect that also heightens injury susceptibility [34]. Previously, Buchheit et al. [41] showed that most players arrived at the match without having been exposed to near-to-maximal sprinting speed running bouts during the training days of the current microcycle. However, while association did not imply causation in this research, match hamstring injuries in elite soccer were systematically lower when > 95% maximal sprint exposures were performed during training two days prior to a match [41]. Although, this specific topic warrants further research, since chronic data was not associated with non-contact muscle injuries in the present study.

To the authors knowledge, this is the first study to analyze the impact of acute and chronic loads separately. For instance, previous research included metrics such as total distance, HSR distance, very high-speed running distance, and the number of actions performed > 5.3 m/s to calculate ACWR [13]. This study found that injuries appeared when players achieved a mean ACWR of 1.02 au, which is below the “danger zone” (i.e., > 1.5 au) [13]. When compared to the results of the present study, no examined metric achieved such value and only ACWR of peak speed achieved 0.96 au which suggests that ACWR may not be sensitive enough to detect injuries as mentioned previously [42, 43]. In support of the current methodological approach, it is worth highlighting that Bache-Mathiesen et al. [44] recently argued that modelling acute and chronic training loads separately within regression models offers a robust and intuitive framework for analyzing the relationship between relative training load (e.g., RPE-based load) and injury risk. The present study contributes to the growing body of evidence suggesting that analyzing acute and chronic loads as independent variables may be an effective and transparent method for injury risk assessment in elite soccer environments.

Some studies that employed the ACWR lacked a conceptual framework as the ratio violates mathematical premises regarding the construction of the ratios by introducing noise and statistical artifacts, while research intended to be predictive, however was only found to be descriptive [42, 43]. Even when this ratio utilized measures of running distance, contrasting results were found [42, 43]. When the ACWR of HSR distance was above 1.18, this demonstrated a higher injury risk (OR = 1.71) [43]. However, Bowen et al. [45] showed a high overall injury risk for total distance (ACWR above 2.14), although mainly for non-contact muscle injuries and a very high overall risk for distances covered at low-intensity (ACWR above 2.15). Furthermore, Enright et al. [46] analyzed that nearly all of the 142 non-contact injuries investigated in European league teams occurred when players were within the “sweet spot” zone (0.8–1.3 au).

Considering previous literature, evidently the present study found injuries when the ACWR values were much lower than previous studies [43, 45, 46]. Nonetheless, one study [13] reported that eight players were injured when the ACWR was below 0.8 au which implies that a sudden discontinuation of chronic loads with high physical demands would be conducive to an increased risk of injury [42]. Ribeiro-Alvares et al. [13] suggested that a gradual physical de-conditioning process rendered players susceptible to the high match demands while, a deliberate de-load week following several weeks of excessive load might not be adequate for recovery. The same authors suggested that removing players with ACWR below 0.8 from matches could be reasonable to avoid injury [13]. However, the data for the present study showed that the ACWR of total distance, HSR and sprint distance (0.25, 0.25 and 0.25 au for injury players and 0.28, 0.28 and 0.29 au for non-injury players, respectively) presented values lower than 0.8 au which in this case would suggest that no players would be available for the next match. This notion supports the present analysis that emphasized the importance of acute load of HSR distance, sprint distance and peak speed rather than using other calculations such as ACWR.

### Practical applications

These findings highlight the importance of structured load management strategies to mitigate injury risk when players are subjected to sustained high training/match loads. The present data supports the notion that higher volume of HSR and sprint distance in the previous 7-day period significantly reduces the likelihood of noncontact muscle injuries, which consequently supports the exposure of high loads of these metrics on a weekly basis. However, it is pertinent to determine when during the previous 7-day period is most appropriate to achieve the maximum velocity exposure to maximize performance and minimize non-contact muscle injury risk. Some research has suggested that two to three days prior to the match is ideal [8, 41]. Notwithstanding, the results of the present study could provide greater explanation with a longitudinal analysis. For instance, a previous three-year study suggested that acute, excessive and rapid increases in load may be associated with noncontact muscle injuries, rather than chronic exposure to higher loads [45] which may have been prevalent in the current study. Finally, the present study highlighted a value of 87,942 m for C-TD to avoid injury risk. While no other study was found to provide an identical value, only one other study suggested an acute value lower than 21,900 m to reduce non-contact muscle injury risk [47]. Thus, when considering four accumulative weeks for that study [47], the C-TD would be 87,600 m, which is a similar value when compared to the present study (87,942 m). This finding warrants further research to better understand the present values.

### Limitations and future research recommendations

Despite these insights, the study has some limitations. Data was only collected from starting players over a single season, reducing the number of observed non-contact muscle injuries, which may impact statistical power and generalizability. Non-starting status can lead to varying acute and chronic load through various training cycles and as such, these players are physically conditioned and managed differently. In turn, this can have significant practical applications on how coaches and fitness practitioners manage load and prepare starting and non-starting players differently during training cycles. Similarly, the study design did not include a longitudinal analysis to understand the load variation across a season and between seasonal phases, including spikes that may be associated with noncontact muscle injuries, rather than chronic exposure to higher loads as previously suggested [45]. For young soccer players, other contextual factors such as age, playing position, seasonal variations, concealment of injury, imbalance between external pressure and internal effort, training methods, recovery time, can also lead to different injury incidences [48] and should be considered for future research. Additionally, multi-season datasets and longitudinal designs to enhance the robustness of these findings would be beneficial and enhance the existing body of knowledge. Moreover, only running distances and speeds were considered, which did not account for gym-based training and therefore, future studies may include this type of information to improve the contextual environment and analysis. Furthermore, player injury history was not available and therefore was not considered in the analysis and no internal load data (e.g., rating of perceived exertion, heart rate) was collected, which may assist in providing a more holistic perspective and contextualize acute and chronic loads with external load metrics. When considering the novel statistical analysis approach, some limitations should also be noted. Synthetic minority over-sampling technique may introduce synthetic instances that do not accurately represent the minority class, potentially leading to overfitting and unreliable results in real-world medical scenarios [49]. Instead, exploring alternative approaches such as Ensemble Learning-Based Methods like XGBoost and Easy Ensemble which have shown promise in mitigating bias and providing more robust performance can be considered. Collaborating with data science specialists and medical professionals to design and validate these techniques is essential to ensure reliability and effectiveness in medical applications [49]. The data should also be interpreted with caution as it only reflects one team across one season in which only 12 injuries were analyzed. A final recommendation for future studies would be to calculate acute:chronic workload ratio or the exponentially weighted moving average to analyze any relationship with muscle injuries [50] employing an identical study design.

## CONCLUSIONS

In conclusion, this study highlighted that a higher acute load of HSR and sprint distance in the previous 7-day period significantly reduces the likelihood of non-contact muscle injuries. Moreover, excessive exposure to peak speeds within a short acute 7-day period appears to elevate non-contact muscle injury risk, likely due to increased neuromuscular demand and fatigue. Finally, a high chronic load of total distance was identified as a risk factor, with an odds ratio above 1, indicating a potential overload effect that heightens injury susceptibility.
